# IRSOM, a reliable identifier of ncRNAs based on supervised self-organizing maps with rejection

**DOI:** 10.1093/bioinformatics/bty572

**Published:** 2018-09-08

**Authors:** Ludovic Platon, Farida Zehraoui, Abdelhafid Bendahmane, Fariza Tahi

**Affiliations:** 1 IBISC, Université Evry, Université Paris-Saclay, Evry, France; 2 Institute of Plant Sciences Paris-Saclay, INRA, CNRS, Université Paris-Sud, Université d’Evry, Université Paris-Diderot, Orsay, France

## Abstract

**Motivation:**

Non-coding RNAs (ncRNAs) play important roles in many biological processes and are involved in many diseases. Their identification is an important task, and many tools exist in the literature for this purpose. However, almost all of them are focused on the discrimination of coding and ncRNAs without giving more biological insight. In this paper, we propose a new reliable method called IRSOM, based on a supervised Self-Organizing Map (SOM) with a rejection option, that overcomes these limitations. The rejection option in IRSOM improves the accuracy of the method and also allows identifing the ambiguous transcripts. Furthermore, with the visualization of the SOM, we analyze the rejected predictions and highlight the ambiguity of the transcripts.

**Results:**

IRSOM was tested on datasets of several species from different reigns, and shown better results compared to state-of-art. The accuracy of IRSOM is always greater than 0.95 for all the species with an average specificity of 0.98 and an average sensitivity of 0.99. Besides, IRSOM is fast (it takes around 254 s to analyze a dataset of 147 000 transcripts) and is able to handle very large datasets.

**Availability and implementation:**

IRSOM is implemented in Python and C++. It is available on our software platform EvryRNA (http://EvryRNA.ibisc.univ-evry.fr).

## 1 Introduction

Non-coding RNAs (ncRNAs) are transcripts that do not encode for proteins, contrary to coding RNAs. They are of different classes, including the well-known ribosomal RNAs (rRNAs), transfer RNAs (tRNAs) and microRNAs (miRNAs). They play important roles in a wide range of biological processes, and are studied more and more due to their impact in many diseases such as cancer (Bartonicek *et al.*, 2016).

There are multiple tools to discriminate coding and ncRNAs (Fan and Zhang, 2015; Hu *et al.*, 2017; Kang *et al.*, 2017; Kong *et al.*, 2007; Lertampaiporn *et al.*, 2014; Li *et al.*, 2014; Lin *et al.*, 2011; Sun *et al.*, 2013; Ventola *et al.*, 2017; Wang *et al.*, 2013). A basic idea to separate coding and non-coding transcripts is to identify if the transcript code for a protein. The most used feature aside the sequence composition is thus the Open Reading Frame (ORF) size and coverage (Housman and Ulitsky, 2016). The most popular tool, named CPC (Kong *et al.*, 2007), is based on this idea. The authors built a model based on an SVM algorithm using several sequence features like ORFs quality, and on BLASTX results against a protein database. But this approach has an important drawback of time consuming. Recently, a new version of CPC, that is alignment-free, has been proposed by the same group. The new method, called CPC2 (Kang *et al.*, 2017), uses SVM technique to build a model from four features: the Fickett score, the length, integrity and isoelectric point of the longest ORF. CPAT (Wang *et al.*, 2013), CNCI (Sun *et al.*, 2013) and PLEK (Li *et al.*, 2014) are three other existing methods that are alignment-free. CPAT uses four sequence features: the length and coverage of the maximal ORF, the Fickett score (representing the bias of base position in the codons and the percentage composition of each base) and the Hexamer score (log-likelihood ratio between the frequencies of each Hexamer in the sequence with a model computed on coding sequences). The authors built their model with a logistic regression, but the user needs to define a threshold to determine the limit between coding and non-coding transcripts. CNCI and PLEK are both based on sequence motifs and on SVM to build the model from the computed features. CNCI uses the frequency of adjoining nucleotide triplets to find most-like coding DNA sequences (MLCDS). Five features are extracted from the MLCDS, such as sequence codon bias and Sequence-score (S-score). PLEK bases its prediction on an improved *k*-mer scheme where the frequencies are weighted by the *k*-mer length. Note that among these existing tools, only CPAT can be retrained. The sources of CNCI and CPC2 cannot be used to train a model. The available models for CNCI are built only for vertebrates and plants, and the ones for CPC2 are built from a mix of data (Human data for the coding and data coming from GENCODE for the non-coding). PLEK offers a script to train a new model but it is very slow (at least 2 days for a training on a dataset of 7500 sequences with 10 threads).

In this paper, we present IRSOM, a new alignment-free method for discriminating non-coding and coding RNAs. IRSOM is a supervised classifier composed of a Self-Organizing Map (SOM) and a perceptron layer which is fully connected to the SOM. IRSOM uses several features that are related to the sequence statistics (*k*-mers motifs frequencies, codon position biases, nucleotide frequencies and GC content) and the putative ORFs (coverage of the longest ORF, ORFs coverage distribution, start and end codon distribution, ORF frequency, ORF length and the frame bias).

We also associated a rejection option to IRSOM. The rejection allows to keep reliable predictions and to abstain in the situations where the predictions are unreliable. The first work on rejection in machine learning has been proposed in (Chow, 1970) where Chow defines what we call actually the Chow rule. This rule states that a prediction is rejected if its probability is lower to a given threshold. In (Ishibuchi and Nii, 2000), the authors propose two rejection options for neural networks which are the distance rejection option and the ambiguity rejection option. The distance rejection rejects the predictions that are far from the predicted classes while the ambiguity rejection rejects the predictions that are in overlapping areas between two or more classes. The distance rejection option rejects a prediction if the largest value provided by the output neurons is lower than a certain threshold. The ambiguity rejection option rejects a prediction if the difference between the two largest values provided by output neurons is lower than a certain threshold.

In IRSOM, we use the ambiguity rejection in order to identify the ambiguous transcripts that are on the boundaries between the coding and the ncRNAs. Moreover, by combining the rejection option with the SOM, we are able to visualize and analyze the rejected transcripts. For example, analyzing the ORF features profiles in the SOM allows to highlight the known differences between the coding and the ncRNAs and also shows the ambiguous characteristics of the rejected transcripts.

IRSOM was tested on datasets of several and different species (Human, Mouse, *Arabidopsis thaliana*, Zebrafish, *Escherichia coli*, *Saccharomyces cerevisae* and *Drosophila*), and compared to different existing tools: CPC2 (Kang *et al.*, 2017), CPAT Wang *et al.*, 2013), CNCI (Sun *et al.*, 2013) and PLEK (Li *et al.*, 2014). It shows better or equivalent performances than the other tools in prediction results and comparable time consumption with the fastest tools (CPAT and CPC2). It gives an accuracy greater than 0.95 for almost all species, and reaches for some of them more than 0.99. It also demonstrates its capacity to handle very large datasets thanks to its low running time in prediction as well as in training (respectively, less than 2 min and less than 4 min on a Human dataset). In addition, IRSOM is able to visualize the data repartition into clusters for both the coding and the ncRNA classes and to analyze the rejected classifications. The cluster profiles for each feature can also be visualized and analyzed thanks to IRSOM.

The paper is organized as follows. In the next section, we describe our tool IRSOM as well as the used features. After that we describe the datasets and the protocol used to compare our method to the state-of-art tools. Finally, before concluding, we present and discuss in the Results Section the cross-validation and prediction results, as well the running time we obtained with IRSOM and the different tested tools. In the same section, we show also the interest of the rejection option in a study case.

## 2 Materials and methods

In this section, we describe our algorithm IRSOM for discriminating coding and ncRNAs. IRSOM is a improvement of our previous work in (Platon *et al.*, 2017). IRSOM is based on an original supervised SOM approach including a reject option, that we propose in order to well classify the transcripts that are clearly defined as coding or ncRNAs and to reject (for further analysis) the ones that are ambiguous. In the following, we present first the SOM approach and then we describe in details our algorithm.

### 2.1 Self-organizing map

SOM (Kohonen, 2001) is a neural network that is able to cluster and visualize high dimensional data. By using an unsupervised competitive learning algorithm, SOM is able to produce a map representing the input space. The produced map can have different structures but the most common one is a grid. Topology of the input space is preserved by using a neighbourhood function during the learning phase of the SOM.

Let be an input dataset *X* such that $x_{i}\in X$ is the feature vector of the *i*th input data. We can define a SOM composed of *U* neuron units such that each neuron unit *u* is a cluster represented by a weight vector $w_{u}\in R^{m}$ such that $w_{u}=[w_{u,1},w_{u,2},...,w_{u,m}]$.

The learning algorithm of SOM is composed of two steps which are:

- Assignation: the Best Matching Unit (BMU) of a given input *x_i_* is computed as follows:
  (1) $$\text{BMU}(x_{i})={\text{argmin}}_{u\in U}∥w_{u}-x_{i}∥$$
  where ∥.∥ represents the L2-norm.
- Update: the BMU and its neighbours are updated toward *x_i_* by:
  (2) $$w_{u}(t+1)=w_{u}(t)+\text{α}(t)h_{t}(\text{BMU}(x_{i}),u)(x_{i}-w_{u})$$
  where α(t) is the learning rate and $h_{t}(\text{BMU}(x_{i}),u)=\text{exp}(-\frac{d{\left(\text{BMU}(x_{i}),u\right)}^{2}}{r\times \left(1-\frac{t}{T}\right)})$ is the neighbourhood function (*r* is the radius of the map and *T* is the maximal number of iterations) with $d(\text{BMU}(x_{i}),u)$ the Manhattan distance between the winning neuron and the neuron *u* in the SOM structure.

### 2.2 IRSOM algorithm

**Fig. 1.**
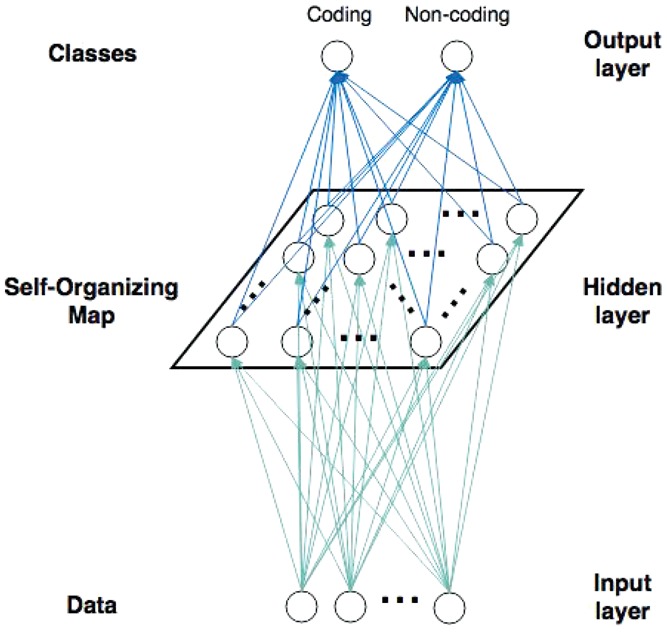
IRSOM architecture

IRSOM is a three layers neural network (Fig. 1) composed of an input layer that represents the input data, a hidden layer which corresponds to a SOM (Kohonen, 2001) and an output layer (supervised layer), that consists of two perceptrons which are fully connected to the neurons of the SOM with forward connections. The SOM computes a new representation of the transcripts using a map of neurons. The perceptron layer is able to assign correctly a class to a transcript by using its new representation. A backpropagation modifies the SOM organization, in order to match with the classes of the transcripts. Moreover, we extend the perceptron layer with a rejection option where the ambiguous predictions are rejected. This rejection option highlight the transcripts which are between the coding and the ncRNAs.

Let a set of input data $X=\{x_{1},x_{2},...,x_{n}\}$ and their corresponding labels $Y=\{y_{1},y_{2},...,y_{n}\}$ such that $y_{i}\in {\left\{0,1\right\}}^{2}$ is a vector representing the label of *x_i_.* An element $y_{i}\in Y$ is defined such that:

(3) $$y_{i}=\{\begin{array}{ll} \left[1,0\right] & \text{ for coding RNAs}\\ \left[0,1\right] & \text{ for non-coding RNAs} \end{array}$$

#### 2.2.1 Learning step

The learning of the weights is divided in two parts, the forward propagation and the backpropagation. During the forward propagation step, the activation of the neurons in the different layers is propagated in order to compute the output and its related errors. The errors are then back propagated during the backpropagation step using gradient descent in order to update the network weights.

##### Forward propagation

In this phase, at each iteration (one iteration corresponds to one batch), the units activations are propagated through the network to generate the output values. These outputs are then compared to the input classes and the error is computed.

In order to keep the organization property of the map, the activation *a_iu_* of the unit *u* depends on *u* and its neighbours u′ such that:

aiu=∑u′∈Uexp(−12∥xi−wu′∥2)σt(u′,u)σt(u′,u)=exp(−d(u′,u)2α×(1−tT)×r)

where d(u′,u) is the Manhattan distance between the neuron u′ and *u* and α is a constant. The output $o_{l}\;\forall l\in \{0,1\}$ of the two perceptrons are computed as:

(4) $$o_{il}={\text{sig(act}}_{il})$$

where ${\text{act}}_{il}=\sum_{u}w_{ul}^{\text{out}}a_{iu}+b_{l}$ and sig is the sigmoid function. $w_{ul}^{\text{out}}$ is the connection between the perceptron *l* and the map unit *u* and *b_l_* is the bias of the perceptron *l.*

The sigmoid function is defined by:

(5) $${\text{sig(act}}_{il})=\frac{1}{1+\text{exp}(-{\text{act}}_{il})}$$

We use a loss function L(), which consists of the cross-entropy cost function C() and a L2-norm regularization term. The regularization aims to improve the generalization of a learned model and to avoid overfitting.

(6) $$L(Y,O)=C(Y,O)+\text{λ}\sum_{u}∥w_{u}^{\text{out}}{∥}^{2}$$

where *O* is a vector containing the output of the perceptrons, λ is the parameter which controls the importance of the regularization term and

(7) $$C(Y,O)=-\frac{1}{N}\sum_{i}\sum_{l}y_{il}\ln\left(o_{il}\right)$$

##### Backpropagation

This phase allows to calculate the error contribution of each unit after a batch is processed. The gradient descent optimization algorithm is used to adjust the output weights and the SOM weights by computing the gradient of the loss function *L*(*Y*, *O*). After the computation of the error, it is distributed back through the layers of the supervised SOM network. The weights of our neural network are optimized using the momentum optimizer such that:

(8) $$w(t+1)=w(t)-{\text{μ}}_{1}\times \left({\text{μ}}_{2}\times {\text{acc}}_{w}+\frac{\partial L(Y,O)}{\partial w}\right)$$

where *w* is a weight of the neural network, acc*_w_* represents the sum of the gradient for this weight over the iterations and μ_1_ and μ_2_ are constants controlling, respectively, the learning rate and the importance of the accumulation.

And the gradients of the output weights is given by:

∂L(Y,O)∂wulout=−1N∑i∑lyil×1oil×sig(actil)×(1−sig(actil))×aiu+2λwulout

The gradient of the SOM weights is computed by:

∂L(Y,O)∂wu=−1N∑i∑lyil×1oil×sig(actil)×(1−sig(actil))×σt(BMU(xi),u)×(xi−wu)×exp(−12∥xi−wu∥2)

#### 2.2.2 Prediction step

During the prediction, we compute the output of the perceptrons using an activation function slightly different. For a given unit, the activation does not rely on its neighbors. We compute the activation $a\prime_{iu}$ of the unit *u* for the transcript *x_i_* by:

(9) $$a_{iu}=\text{exp}\left(-\frac{1}{2}∥x_{i}-w_{u}{∥}^{2}\right)$$

The activation of the unit *u* depends only on itself and the input *x_i_* because during the prediction step we assume that the training step is finish. So the effect of the neighbour in the activation function is null (${\text{σ}}_{t}(u\prime ,u)=0$ for u≠u′).

The output of the perceptrons $o_{l}\;\forall l\in \{0,1\}$ is computed as follows:

(10) $$o_{il}=\text{sig(act}\prime_{il})$$

where $\text{act}\prime_{il}=\sum_{u}w_{ul}^{\text{out}}a\prime_{iu}+b_{l}$.

The class of a transcript is determined by the maximal output of the perceptrons:

(11) $$\text{class}(x_{i})=\{\begin{array}{ll} \text{coding RNA } & \text{ if }o_{i0}>o_{i1}\\ \text{non coding RNA } & \text{ otherwise} \end{array}$$

#### 2.2.3 Reject option

To improve the reliability of our method and identify the ambiguous transcripts, we use one of the rejection approaches proposed in (Ishibuchi and Nii, 2000). The greater the difference between $o_{i0}$ and $o_{i1}$ is, the greater is the confidence in the prediction. By following the second rejection method in the article (Ishibuchi and Nii, 2000), we can improve the reliability of the prediction by rejecting the ambiguous classifications. We are able to define a classifier with rejection option called $\text{ψ}(x_{i})$ such that:

(12) $$\text{ψ}(x_{i})=\{\begin{array}{ll} -1 & \text{ if }|o_{i0}-o_{i1}|<\text{β}\\ \arg\max_{l}\;o_{il} & \;\text{otherwise} \end{array}$$

where β is the rejection threshold. When the absolute difference value between $o_{i0}$ and $o_{i1}$ is lower than a threshold β, the prediction is rejected and set to –1.

The parameter β is application dependent. For certain applications we may want a high β in order to have the most reliable predictions but in an exploratory analysis, we may use a smaller β in order to keep more predictions even if they are potentially misclassified.

### 2.3 Features

Our IRSOM algorithm is an alignment-free method based on three types of features which are sequence bias, ORF statistics and *k*-mer motifs:

- Sequence bias: composed of three features, which are the codon position bias, the frequencies of each nucleotide and the GC frequency. The purpose of the codon position bias is to measure if there is nucleotide position bias in codons. It is computed as follows:

$$X_{\text{pos}}=\frac{\text{min}(X_{1},X_{2},X_{3})}{\text{max}(X_{1},X_{2},X_{3})}$$

where for a given base *X*: *X*_1_ is the number of *X* in positions 0, 3, 6, …; *X*_2_ is the number of *X* in positions 1, 4, 7, … and *X*_3_ is the number of *X* in positions 2, 5, 8, ….

- ORF: we compute the length and coverage of the maximal ORF that are useful to access the information of the most probable coding sequence of the transcript. In order to rescale the ORF length, we defined the transformed ORF length such that:

$$\text{ORF length}={\text{log}}_{10}\left(x\right)$$

where *x* is the raw ORF length.

We consider the mean and SD of the length and coverage of all the possible ORFs. Moreover, we compute the mean and SD of the start and end codon of all the possible ORFs in the transcript. We add also in our model the frame bias of the ORFs and the ORF frequency such that:

Frame bias=1−mini∈{0,1,2}|ORFi|maxi∈{0,1,2}|ORFi|ORF frequency=∑i∈{0,1,2}|ORFi|Number of start codon

where ORF*_i_* is the ensemble of ORFs in the frame *i.*

- *k*-mer: the *k*-mers are all the words of size K that are contained in a string. Here, we select the *k*-mers of size 3, 4 and 5 and compute their frequencies.

### 2.4 Implementation

IRSOM is composed of two major parts, the computation of the features and the neural network. The neural network is implemented using TensorFLow which is an open-source software library for machine learning supported by Google (https://www.tensorflow.org/). This library is available for the three major operating systems (Windows, Ubuntu and macOS) and also for other systems like Android or iOS. The strength of TensorFlow is its capacity to use CPU or GPU without change in the source code, its scalability to big data and the possibility to use HPC clusters.

**Fig. 2.**
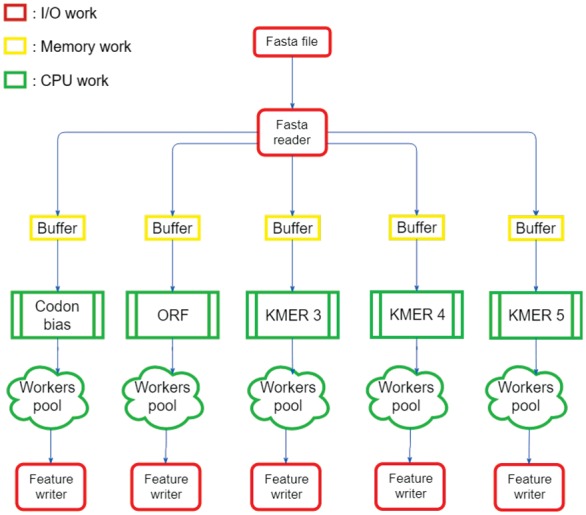
Workflow computation features architecture

The computation of the features is achieved by a workflow API that we developed in C++. The workflow (Fig. 2) is composed of five worker pools which are pools of threads. This workflow is designed to be scalable to big data by using a system of buffer. The use of buffer helps to manage the memory consumption such that the memory is used only for the data that can be processed.

The combination of our API workflow and the use of TensorFlow allows to ensure low computing times and then to have a tool able to handle large datasets.

## 3 Results

### 3.1 Datasets

**Fig. 3.**
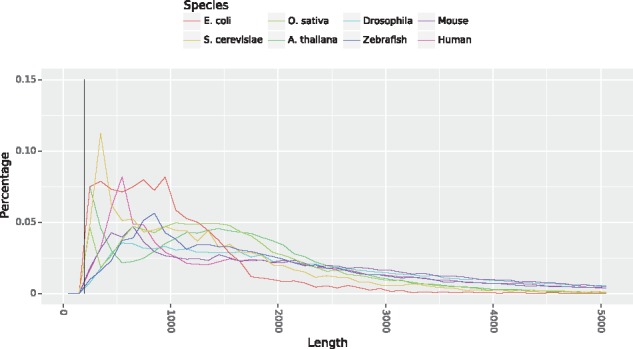
Distribution of transcripts lengths for all considered datasets. The black vertical line separates the transcripts smaller than 200 from the longest ones

We evaluated our method with coding and ncRNAs coming from several species. In order to cover a large spectrum of species from different reigns, we selected RNAs from Human, Mouse, *Oryza sativa*, *A. thaliana*, Zebrafish, *E. coli*, *S. cerevisae* and *Drosophila.* The number of transcripts and their origins are given in Table 1 and the distribution of the transcripts length is shown in Figure 3.

**Table 1.** Benchmark datasets

Species	Coding		Non-coding	
	Origin	Number	Origin (RNAcentral)	Number
Human	Ensembl 92	45 956	Gencode	30 171
Mouse	Ensembl 92	23 715	Gencode	17 582
Zebrafish	Ensembl 92	41 760	Rfam	13 885
*O. sativa*	Ensemble plants 38	42 362	Rfam	6076
*A. thaliana*	Ensemble plants 38	19 228	Rfam, RefSeq, TAIR	7036
*S. cerevisiae*	Ensembl 92	6684	Rfam	1355
*Drosophila*	Ensembl 92	13 928	Flybase	3610
*E. coli* (K-12)	Ensembl bacteria 37	4083	Rfam	1058

The sequences of coding transcripts were extracted from the Ensembl databases (Zerbino *et al.*, 2018). We selected only the transcripts available in the Swiss-Prot database (Consortium, 2017b) in order to have manually curated transcripts except for the Zebrafish and the *Drosophila.* Due to their low amount of transcripts we select the transcripts with ID in the UniParc. The non-coding transcripts come from the RNAcentral database (Consortium, 2017a). RNAcentral combines the information of multiple ncRNA databases [Ensembl (Zerbino *et al.*, 2018), Rfam (Kalvari *et al.*, 2017), RefSeq (O’Leary *et al.*, 2016), GENCODE (Derrien *et al.*, 2012)].

In the case of the Human and Mouse datasets, we selected the ncRNAs coming from the GENCODE database due to their manual curation. For the *Drosophila* dataset, we selected the ncRNAs available in the Flybase database which is a reference database for the *Drosophila.* For the *A. thaliana*, we got transcripts from three databases in order to have enough data: Rfam, RefSeq and TAIR (Berardini *et al.*, 2015; a database specialized on *A. thaliana*). Finally, for the other datasets, we selected the transcripts coming from the Rfam database.

### 3.2 Experimental protocol

We show the performance of our tool by comparing it to four classical ncRNA identification tools which are CPAT (Wang *et al.*, 2013), CPC2 (Kang *et al.*, 2017), CNCI (Sun *et al.*, 2013) and PLEK (Li *et al.*, 2014).

The benchmark was executed on a 50 cores virtual machine (VM) under debian with 128 Gb available memory. Each tool was launched separately on the VM with the sequences in fasta format.

We performed two types of performance analysis: a cross-validation analysis and a prediction analysis. For this purpose, each of the different datasets presented above, noted *D*, has been divided into two subsets (of same size) $D_{\text{sub}1}$ and $D_{\text{sub2}},\;D_{\text{sub1}}$ used in the cross-validation and $D_{\text{sub2}}$ in the prediction.

In the cross-validation, we evaluate our tool IRSOM in order to measure the impact of the rejection threshold on the different datasets. We therefore performed a 10-fold cross-validation with IRSOM, and then a prediction with the different tools, CPAT, CPC2, CNCI, PLEK and IRSOM.

Among the tested tools, only CPAT was retrained on all datasets. Unfortunately, the sources to build a new model for CPC2 and CNCI are not available and PLEK is too slow to create new models (more than 2 days for a training on a dataset of 7500 sequences with 10 threads). We did not run CNCI on the datasets of the *Drosophila*, *E. coli* and *S. cerevisae* because the available models were designed for vertebrate or plant organisms only. PLEK authors built their model with a Human dataset but in their article (Li *et al.*, 2014), they show results on vertebrate species. We then run PLEK on the vertebrates datasets. Finally, IRSOM and CPAT are trained on each of the considered species, as well as on all species together (cross-species model). In this last case we note the two tools as IRSOM_cross and CPAT_cross, respectively.

The default parameters of the tested tools have been used during the training and prediction as explained by the authors in their respective documentation. For each model computed with CPAT, we determined the threshold separating the coding and the ncRNAs by maximizing the sensitivity and specificity. For PLEK, we set the minimal length of an accepted transcript to 20 in order to keep the small transcripts (which represent a small part of the datasets).

In case of our tool, there are different parameters. The SOM dimension which is a grid of size 10 × 10. The parameter α in the σ function is set to 0.5. The regularization factor is set to 0.001. The size of the batches is set to 100. The learning constants μ_1_ is set to 0.05 and μ_2_ is set to 0.25. The upper limit of the number of iterations is set to 10 000, but the training step can end earlier if the difference between the loss function of two consecutive iterations is smaller than $10^{-6}$. The values assigned to the different parameters are computed on the training set used for the cross-validation. We initialize the SOM in the hidden layer by the SOM learning algorithm.

### 3.3 Results

We measure the classification performance using three measures:

- Accuracy: represents the percentage of correctly classified RNAs, it is defined as follows:

(13) $$\text{Acc=}\frac{\text{TP+TN}}{\text{TP+FP+FN+TN}}$$

- Sensitivity: measures the rate of true positives:

(14) $$\text{Sensitivity=}\frac{\text{TP}}{\text{TP+FN}}$$

- Specificity: measures the rate of true negatives:

(15) $$\text{Specificity=}\frac{\text{TN}}{\text{TN+FP}}$$

where TP are the true positives, TN are the true negatives, FP are the false positives and FN are the false negatives. Here, the positive class represents the ncRNAs and the negative one the coding RNAs. In the case of IRSOM, the TP, TN, FP and FN are computed on the non-rejected data.

#### 3.3.1 Cross-validation results

**Fig. 4.**
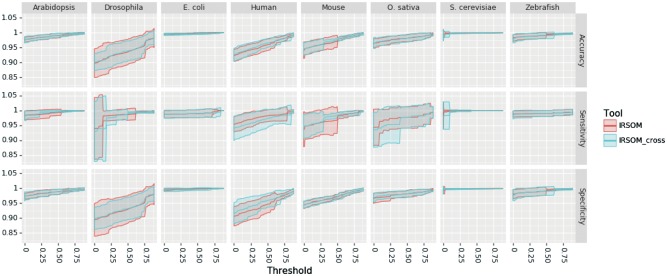
IRSOM performance (mean ± SD) in regard to the rejection threshold for all the datasets

As mentioned above, we performed a 10-fold cross-validation with our tool IRSOM. For each species dataset *D*, we applied IRSOM on the subset $D_{\text{sub1}}$ (as described in Section 3.2). The obtained results are given in Figure 4 and show the performance of IRSOM for different rejection thresholds.

**Fig. 5.**
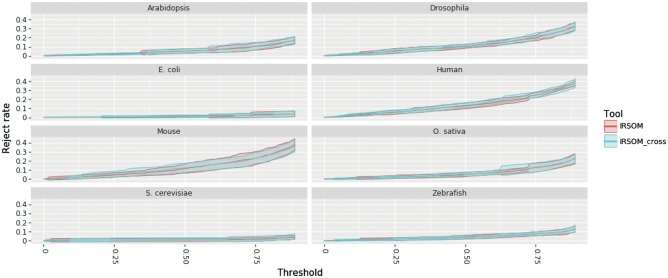
IRSOM reject rate (mean ± SD) in regard to the rejection threshold for all the datasets

As we can see on the Figure 4, the performance of IRSOM increases when we increase the rejection threshold. But as we can see in Figure 5, the number of rejected prediction increases when the rejection threshold increases.

In order to define a good rejection threshold, we need to find a trade off between the performance and the reject rate. For the cross-species model of IRSOM, we set the rejection threshold at 0.7. This threshold gives good performance for all species (Accuracy greater than 0.975) with a reasonable rejection rate (less than 20% for all species). For the species specific model, we set a threshold for each species. We set the thresholds to values that show the highest performances. For the *S. cerevisiae* and *E. coli*, we set a threshold of 0.1 and 0.2, respectively. For the plants, we set a threshold of 0.6 for the *Arabidopsis* and 0.8 for the *Oryza sativa.* The eucaryotes species have a wider range of threshold. We set a threshold of 0.5 for the Zebrafish, 0.75 for the *Drosophila* and Mouse and 0.8 for the Human. For all the species, we have at most 30% of the predictions that are rejected. For most of them, we have a reject rate lower than 20% (*A. thaliana thaliana* and *Oryza sativa*) or even 10% (*E. coli*, Zebrafish and *S. cerevisiae*). It has to be noticed that the thresholds and rejection rate increase with the complexity of the species. This variation can be due to the potential higher ambiguity between the coding and the ncRNAs in complex organisms.

#### 3.3.2 Prediction results

On each species dataset *D*, we performed predictions on the subset $D_{\text{sub2}}$, using the different tools IRSOM, CPC2, CPAT, CNCI and PLEK. The predictions were processed using the provided models in case of CPC2, CNCI and PLEK and with the models obtained after a training on the subset $D_{\text{sub1}}$ in case of IRSOM and CPAT. With IRSOM and CPAT, we performed two types of tests, one where the corresponding model is used for the considered species, and one where the cross-species model is used for each of the species.

**Fig. 6.**
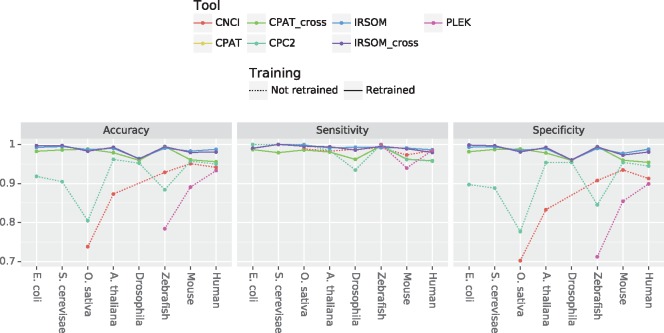
Accuracy results obtained by CNC2, CNCI, CPAT, PLEK and IRSOM on each of Human, Mouse, *A. thaliana thaliana*, *Oryza saliva*, Zebrafish, *E. coli*, *Saccharomyces cerevisiae* and *Drosophila* species. CPAT_cross and IRSOM_cross designate, respectively, CPAT and IRSOM when used with the cross-species model. CPAT and IRSOM are by default used on each species with the corresponding model. Two models for CNCI are available, one for vertebrate and one for plants. CPC2 was trained on Human protein and ncRNA in GENCODE. And PLEK was trained on Human data

Figure 6 shows the obtained results. For IRSOM, we use the rejection thresholds defined previously on the subset $D_{\text{sub1}}$. The tools retrained on our datasets, i.e. IRSOM and CPAT, are represented by full lines and the tools that we could not retrain (we used the provided models) are represented by dotted lines.

**Fig. 7.**
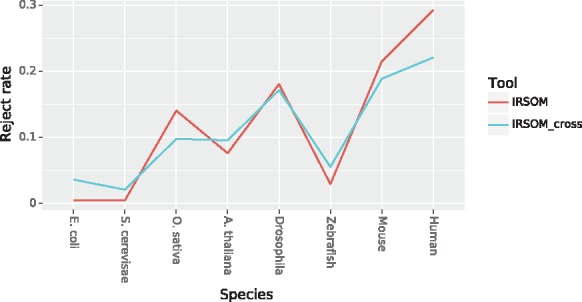
Rejection rate of both IRSOM models on all the datasets

The obtained results show a good performance of our tool IRSOM compared to the other tools. IRSOM exceeds 0.95 in accuracy for all the species. Compared to CPAT, the only tool we succeeded to retrain on our data and the second best tool, IRSOM shows slightly better results for all considered species. Furthermore, the two models of CPAT show the same performance on all datasets as for the two models of IRSOM (except on the Human) but with different reject rate (Fig. 7).

CPC2 shows an accuracy greater than 0.9 on every datasets except on the Zebrafish (0.88) and the *O. sativa* (0.8). These results are explained by the lower specifitciy on these datasets (0.84 for the Zebrafish and 0.77 for *O. sativa*). CNCI was used only on the vertebrates (Human, Mouse and Zebrafish) and plants (*A. thaliana* and *O. sativa*) datasets according to their models. CNCI shows lower results than CPAT, CPC2 and IRSOM on all the datasets, except on the Zebrafish where it gives better results than CPC2.

The tool that gives the worst results is PLEK. As mentioned above, the model provided by the authors is the result of a training on Human dataset. On the Human and Mouse datasets, PLEK shows suitable results. As prospected, the best results are those on Human (0.93). On the Zebrafish, we obtained an accuracy of 0.78 when the authors have shown in (Li *et al.*, 2014) an accuracy of 0.91.

Finally, IRSOM gives the best performances (accuracy mean: 0.98) compared to the others tools [CPAT (both models): 0.97, CPC2: 0.91]. Every tool gives a nearly perfect sensitivity [IRSOM (both models): 0.99, CPAT (both models): 0.97 and CPC2: 0.97] but lower performance in term of specificity except for IRSOM (0.98 for both models) and CPAT (0.97 for both models). These results suggest that all the tools are able to correctly identify the ncRNAs, but predict wrongly a part of the coding RNAs. With our rejection option, we can identify and reject the prediction of the ambiguous transcripts and improve our specificity. Furthermore, the mean reject rate of both models is around 15% with a slightly higher reject rate for the species specific model (Fig. 7). These results confirm the ability of our method to gives accurate prediction and identify the ambiguous transcripts without rejecting too much data. In addition the rejected transcripts can be visualized and analyzed using the SOM.

#### 3.3.3 Study case

**Fig. 8.**
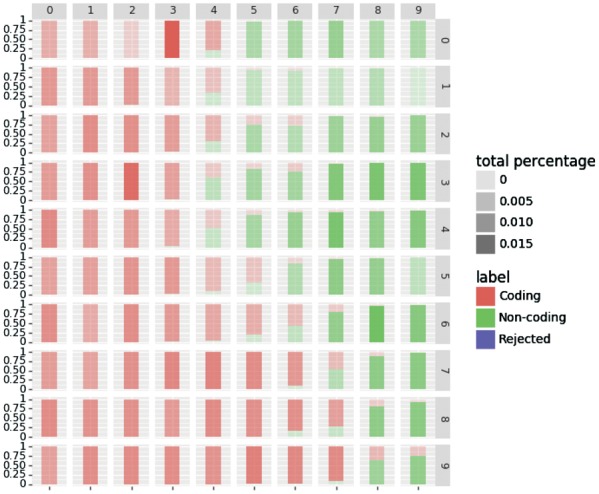
True label distribution in the SOM with the Human dataset

**Fig. 9.**
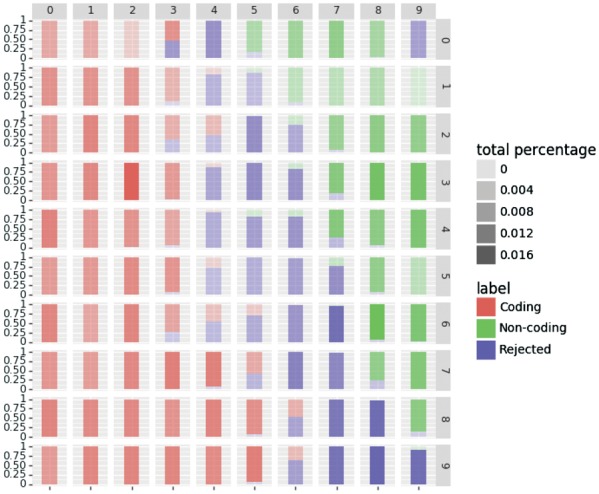
Rejected label distribution in the SOM with the Human dataset

Here, we demonstrate how the rejection option improves the prediction results. To do so, we investigate the prediction by visualizing the SOM prototypes and the distribution of the labels in the SOM. One of the most interesting properties of SOM is its capacity to visualize the data by projecting it to a low dimensional space. By using this property, we can extract the profiles of the transcripts that are close to a neuron prototype by taking its representative. In our case, we look at the transcripts that are rejected in the Human dataset using the species specific model of IRSOM. The Figures 8 and 9 show, respectively, the true label distribution and the predicted label (with rejection) distribution of the SOM in the hidden layer of IRSOM. We can see in Figure 8 that the map separates well the coding RNA from the ncRNA. In Figure 9, we can see that the neurons assigned to the rejected predictions are at the boundary between the coding and the non-coding regions in the map except, for one neuron in the top right corner of the map. In the case of this neuron, the non-coding neuron output is too low and so the difference between the two output values is lower than the threshold used for the Human dataset (which is 0.8). As a reminder, a prediction is rejected if the difference between the coding and the non-coding neurons outputs is lower than a threshold β.

**Fig. 10.**
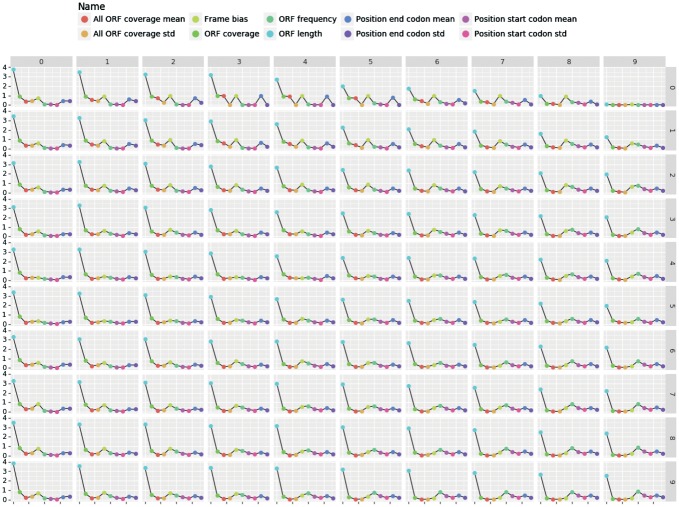
ORF profiles of the neurons for the human dataset where each point represents a value of the ORF feature

The Figure 10 shows the profiles of the representatives for the ORF features. Each point in the figure represents the value of the representative for a given feature. We can see that the coding transcripts have a high ORF coverage while the non-coding transcripts have a lower or near zero ORF coverage as expected. Moreover, the coding transcripts have a low ORF frequency (close to 0) when the non-coding transcripts have a high ORF frequency (close to 1). These features means that for a given number of start codon, there are more ORFs in the non-coding transcripts than in the coding ones.

The characteristics of the transcripts in the rejected area are more ambiguous. They show an ORF coverage of 0.5 with a high ORF frequency like the non-coding transcripts. The average ORF coverage with the high ORF frequency suggests that these transcripts have coding sequences that are not stable as the other coding transcripts. Moreover, these transcripts can potentially produce proteins as the coding RNAs and also have the same function as the ncRNAs.

### 3.4 Running time

To compare IRSOM’s time performance with that of the other tools, we measured the prediction time of each of them on each species dataset (with all the sequences) as well as on the cross-species dataset. The obtained running times on the different datasets, ordered from the smallest to the biggest one, are given in Table 2.

**Table 2.** Prediction running times obtained on a 50-cores VM under debian with 128 Gb of memory and 2.8 Ghz of CPU

Dataset	Dataset size	Running time (in seconds)				
		CNCI	CPAT	CPC2	PLEK	IRSOM
Cross-species	147 322		221	203	1315	254
Human	38 063	2747	60	57	370	82
Zebrafish	27 782	1074	41	36	240	64
*O. sativa*	24 187	520	30	28	202	59
Mouse	20 648	2958	34	30	206	56
*Drosophila*	17 047		39	32	197	51
*A. thaliana*	13 112	316	20	17	120	43
*S. cerevisiae*	3913		7	5	40	29
*E. coli*	2570		4	3	33	27

As we can see in Table 2, our tool IRSOM gives comparable running time compared to the existing tools. For instance, on the Human dataset, which is composed of around 38 000 sequences, it took around less than 2 min for the prediction and also less than 4 min to generate the Human model.

The prediction time difference between IRSOM and CPAT and CPC2 (which are the two fastest) is due to the computation of the features. In our tool, the features are computed by a c++ executable and imported in a python script for the prediction (or training). By doing so, we are able to handle large volumes of data but induce a overhead. In CPAT and CPC2, everything is done in python and so there are no overhead. As we can see, for every dataset, we have a constant difference in running time of 20–30 s with CPAT. However, IRSOM is one of the fastest tool and gives comparable results to CPC2 and CPAT.

## 4 Conclusion

We presented here a new approach and tool for identifying rapidly and efficiently ncRNAs. Our tool, called IRSOM is able to accurately discriminate coding and ncRNAs. Furthermore, with our rejection option, we are able to identify the ambiguous transcripts and analyze them with the SOM. Compared to the state of art, our tool gives the best results on several species of different reigns. It gives also good time computing for small and large datasets.

By using the rejection option, we are able to increase the prediction accuracy. Moreover, we highlight the fact that the limit between coding and non-coding transcripts is not well defined. And so, the coding and the non-coding transcripts have to be seen as a range of transcripts instead of two separable types of transcripts.

One of our future work to improve the rejection part of our algorithm is to define a method to compute the optimal rejection threshold. One way to do this is to follow the Chow method where we compute the optimal rejection threshold by defining the cost of a rejection. An other way would be to define a loss function which is dependent on the rejection such as the one described in (Cortes *et al.*, 2016). A third possibility available only for neural network is to define a neuron in the output network that represent the rejected predictions. This approach needs the definition of a loss function to train the additional neuron. The third approach represent a novelty in the field.

We also intend to extend our algorithm in order to take into account different heterogeneous data sources. The sources could be numerical vectors or more complex data like graphs. By doing so, we will be able to use new features such as secondary structures or epigenetic profiles for the classification task. These new features will be used to classify ncRNAs into different classes corresponding to ncRNA types, like for example transfert RNA (tRNA), ribosomal RNA (rRNA), microRNA (miRNA) or piwi RNA (piRNA).
